# A systematic review of patient‐reported dignity and dignified care during acute hospital admission

**DOI:** 10.1111/jan.15370

**Published:** 2022-07-16

**Authors:** Abdul‐Ganiyu Fuseini, Lenore Ley, Helen Rawson, Bernice Redley, Debra Kerr

**Affiliations:** ^1^ Centre for Quality and Patient Safety, Institute for Health Transformation, School of Nursing and Midwifery Deakin University, Geelong Waterfront Campus Geelong Victoria Australia; ^2^ Nursing and Midwifery, Faculty of Medicine, Nursing and Health Sciences Monash University Melbourne Victoria Australia

**Keywords:** acute care, acute hospital admission, autonomy, communication, dignified care, dignity, healthcare professionals, nursing, patients, privacy, systematic review

## Abstract

**Aims:**

To synthesize quantitative evidence on levels of dignity during acute hospital admission and identify barriers and facilitators to patients' dignity or dignified care from the perspective of hospitalized patients. The secondary aim was to examine the relationship between dignity and demographic, clinical and psychological characteristics of patients.

**Design:**

A systematic review based on the protocol of the Preferred Reporting Items for Systematic reviews and Meta‐Analyses guideline for reporting systematic reviews.

**Data Sources:**

Five electronic databases (PubMed, CINAHL, Embase, PsycINFO, AgeLine) were searched in February 2021, followed by backward‐forward searching using Web of Science and Scopus databases.

**Review Methods:**

Potentially eligible articles were scrutinized by two reviewers. Articles that met the eligibility criteria were appraised for quality using the Critical Appraisal Tool for Cross‐Sectional Studies. Two reviewers extracted data for the review and resolved differences by consensus.

**Results:**

Out of 3052 potentially eligible studies, 25 met the inclusion criteria. Levels of dignity for hospitalized patients vary widely across geographic locations. Patients' dignity is upheld when healthcare professionals communicate effectively, maintain their privacy, and provide dignity therapy. Patients' perceptions of dignity were, in some studies, reported to be associated with demographic (e.g. age, marital status, gender, employment, educational status), clinical (e.g. hospitalization, functional impairment, physical symptoms) and psychological (e.g. depression, anxiety, demoralization, coping mechanisms) variables whilst other studies did not observe such associations.

**Conclusion:**

Patients in acute care settings experience mild to a severe loss of dignity across different geographic locations. Patients' dignity is influenced by several demographic, clinical and psychological characteristics of patients.

**Impact:**

The findings of the review support impetus for improvement in dignified care for hospitalized patients, addressing factors that facilitate or impede patients' dignity. Measures aimed at alleviating suffering, fostering functional independence and addressing patients' psychosocial needs can be used to promote dignity.

## 1 INTRODUCTION

Dignity is a primary concern to hospitalized patients and is important to their well‐being (Bagherian et al., 2020; Woolhead et al., 2004). Maintaining patients' dignity in acute hospital settings is challenging because dignity is a dynamic concept influenced by combinations of demographic, organizational and healthcare professional (HCP)‐related factors (Avestan et al., 2015; Liu et al., 2020). Nurses are bound by professional codes of conduct to uphold the dignity of their patients and to treat them with respect regardless of the challenges they face (International Council of Nurses, 2012; Nursing and Midwifery Board of Australia, 2008).

Dignity is described in the literature from two perspectives (Allard et al., 2018; Gallagher et al., 2012; Jacobson, 2007). The first perspective defines dignity as inherent self‐worth that is inalienable and given to all human beings by virtue of their rationality and ability to act as moral agents (Allard et al., 2018; Hasegawa & Ota, 2019; Jacobson, 2007). This type of dignity, also termed as intrinsic dignity (Allard et al., 2018), human dignity (Jacobson, 2007, 2009), basic dignity (Nordenfelt, 2003) or absolute dignity (Eriksson, 1988), postulates that all human beings are born with an inherent sense of self‐worth by virtue of being human and are equal in rights and value as enshrined in article one of the United Nations (1948) Universal Declaration of Human Rights. The second perspective defines dignity as an acquired sense of self‐worth that is influenced by the individual's interaction with others in society. This type of dignity, also termed as extrinsic dignity (Allard et al., 2018), social dignity (Jacobson, 2007, 2009), dignity of merit (Nordenfelt, 2003, 2004) or relative dignity (Eriksson, 1988), can be measured by others and has the tendency to be lost through an individual's interaction with others in society.

Efforts have been made to clarify the concept of dignified care to serve as an impetus for improvement in care. According to Tauber‐Gilmore, Norton, et al. (2018), dignified care is care which supports, promotes, and does not undermine the self‐worth of the patient regardless of any differences in sociodemographic characteristics between the patient and HCPs. Dignified care has also been described as shared decision making, patient privacy and autonomy and treatment of patients as one would expect to be treated. (Cairns et al., 2013; Lin et al., 2013). The provision of dignified care has evolved from recognition of patients' self‐worth into a complex endeavour that considers the biopsychosocial make‐up of the individual. This systematic review summarizes quantitative evidence on levels of dignity during acute hospital admission and examines barriers and facilitators to dignified care and the relationship between dignity and other factors.

## 2 BACKGROUND

Over the last two decades, there has been a growing body of empirical evidence on patients' dignity or dignified care for hospitalized patients in acute care settings and different population groups (Chochinov, 2002; Gallagher et al., 2008; Martin‐Ferreres et al., 2019). The majority of studies on patient dignity or dignified care have been undertaken amongst patients with advanced cancer (Bagherian et al., 2020; Bovero, Sedghi, Botto, et al., 2018; Vehling & Mehnert, 2014) or palliative care needs (Houmann et al., 2014; Julião et al., 2017; Pringle et al., 2015). Similarly, several other studies have been conducted amongst hospitalized older adults (Fuseini et al., 2022; Šaňáková & Čáp, 2019; Tauber‐Gilmore, Addis, et al., 2018), and patients with heart failure (Amininasab et al., 2017; Salehi et al., 2020). The majority of these studies have also been conducted using qualitative designs with a high level of subjectivity and limitations for generalisability. These studies, however, have revealed that patients hospitalized in acute care settings often feel vulnerable, lack privacy and autonomy (Bláhová et al., 2020; Kerr et al., 2020) and may be experiencing a loss of dignity (Liu et al., 2020; Philipp et al., 2016).

Researchers have synthesized existing evidence on patients' dignity or dignified care to guide improvement in care and inform the direction of healthcare policy (Clancy et al., 2021; Šaňáková & Čáp, 2019; Zahran et al., 2016). The majority of these reviews were conducted using narrative or integrative review designs. These studies have summarized several barriers to dignity or dignified care including poor HCP‐patient communication, lack of privacy, the tension between professional values and organizational demands, lack of involvement in care decisions and having a cognitive impairment. They also have identified facilitators for dignity or dignified care including respectful and empathetic care, patient involvement in care decisions and patient autonomy (Clancy et al., 2021; Nouri et al., 2017).

Two systematic reviews (Martínez et al., 2017; Xiao et al., 2019) have been undertaken to summarize evidence on the effectiveness of dignity therapy (a brief psychotherapy designed to reinforce self‐worth) amongst palliative care cancer patients. The reviews found a significant effect of dignity therapy in improving dignity. Dignity therapy was also found to be effective in ameliorating depression and anxiety (Martínez et al., 2017).

Although studies have synthesized evidence on patients' dignity or dignity care using an integrative review approach, no efforts have been made to synthesize the quantitative evidence on levels of dignity (i.e. extrinsic, social or relative dignity) during acute hospital admission and their relationship with demographic, clinical and psychological variables of hospitalized patients.

## 3 THE REVIEW

### 3.1 Aims

The aim of the review was to synthesize quantitative evidence on levels of dignity during acute hospital admission and identify barriers and facilitators to patients' dignity or dignified care from the perspective of hospitalized patients. The secondary aim was to examine the relationship between dignity and demographic, clinical and psychological characteristics of patients.

### 3.2 Study design

This systematic review was conducted following the approach described by Tawfik et al. (2019). This approach includes 14 stages, namely: research question or objectives, preliminary research and idea validation, inclusion and exclusion criteria, search strategy, searching of databases and exporting of search results, protocol writing and registration, title and abstract screening, full text downloading and screening, manual searching, data extraction and quality assessment, data checking, data analysis, double data checking and manuscript writing, revision, and submission to a journal. Reporting of results of the systematic review was guided by the PRISMA updated guideline for reporting systematic reviews (Page et al., 2021) (see Appendix S1). The protocol was registered with the International Prospective Register for Systematic Review (PROSPERO) (reference CRD42020216808) (National Institute for Health Research, 2020).

### 3.3 Search methods

Relevant databases (CINAHL, Medline, EMBASE, PsycINFo, AgeLine) were systematically searched on 14 February 2021, and updated on 08 June 2022 with the aid of a search planner, developed by the authors with support from a research librarian. The controlled vocabulary of each database was searched to identify Medical Subject Headings (MESH) (e.g. ‘Dignity’, ‘Human Dignity’, ‘Privacy and Confidentiality+’, ‘Patient Outcome Assessment+’, ‘Health Care+’, ‘Patient Satisfaction+’, ‘Cross Sectional*’) and key words (e.g. ‘dignified car*’, ‘dignity with car*’, ‘patient dignity’, Hospital*, Inpatient*). Backward‐forward searching of citations and references of selected articles was also performed using Web of Science and Scopus databases to identify any relevant articles not previously captured in the search. The search strategy is attached as supplementary information (see Appendix S2). The criteria for inclusion of studies were those that quantitatively measured patient dignity or dignified care for hospitalized patients. Details of the inclusion and exclusion criteria for the review are presented in Table 1.

**TABLE 1:** Criteria for selection of articles.

Inclusion criteria	Exclusion criteria
Published in English in a peer‐reviewed journal.	Conference proceedings or abstract‐only papers.
Published between January 2000 and June 2022.	Articles that reported on patients' dignity or dignified care only from the perspectives of family members and/or HCPs.
Articles available in full text with an abstract.	Articles written as opinion or review papers.
A least 50% of the sample were inpatients, and data were analysed separately for each participant group (e.g. inpatients, outpatients, HCPs or family members).	Validation studies without measurement of patient‐reported dignity or dignified care.

### 3.4 Search outcomes

**FIGURE 1:**
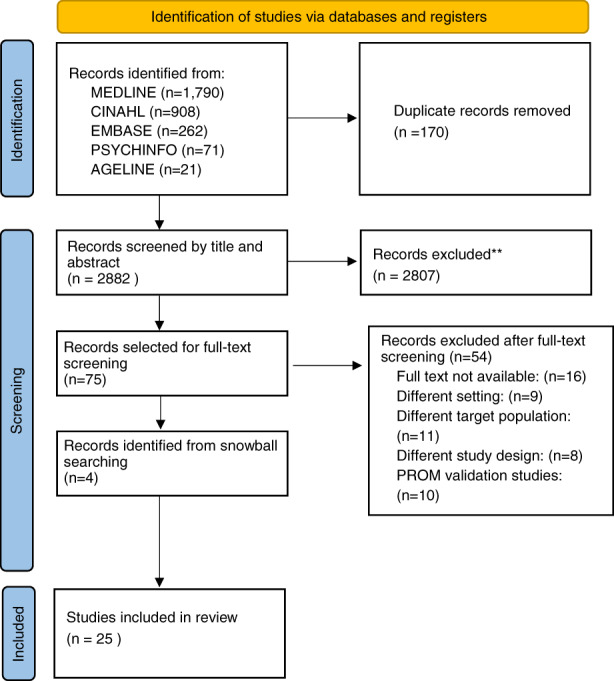
Search results and study selection and inclusion process. From: Page, M. J., McKenzie, J. E., Bossuyt, P. M., Boutron, I., Hoffmann, T. C., Mulrow, C. D., Shamseer, L., Tetzlaff, J. M., Akl, E. A., Brennan, S. E., Chou, R., Glanville, J., Grimshaw, J. M., Hróbjartsson, A., Lalu, M. M., Li, T., Loder, E. W., Mayo‐Wilson, E., McDonald, S., … Moher, D. (2021). The PRISMA 2020 statement: An updated guideline for reporting systematic reviews. The BMJ, 372, n71. https://doi.org/10.1136/bmj.n71.

All included studies (citations and abstracts) were imported into a bibliographic manager (Zotero), and then transferred into Covidence for screening and data extraction. Of 3052 articles identified from the relevant databases, 170 duplicates were removed, before two reviewers independently screened the remaining 2882 articles by title and abstract review, with 2807 articles not meeting the inclusion criteria. The independent full‐text review of the remaining 75 articles by the two reviewers led to exclusion of 54 articles (See Figure 1). Two papers (Bagheri et al., 2018a) and (Bagheri et al., 2018b) presented results from the same cohort of participants, hence were treated as one study in the review. Four additional articles were identified from searching reference lists of included articles. Hence, there were 25 articles included in the review, as shown in the PRISMA flow diagram (Figure 1) (Page et al., 2021).

### 3.5 Quality appraisal

The quality of studies included in the review was assessed using the Critical Appraisal Tool for Cross‐Sectional Studies (AXIS tool) (Downes et al., 2016). The AXIS tool is a 20‐item critical appraisal tool with each item rated on three responses (yes, no and do not know). For every positive response, a score of one is assigned. Higher scores represent a lower risk of bias. Other researchers have used these criteria to differentiate the quality of articles according to the AXIS tool (De Cock et al., 2022; Sujatha et al., 2021). Two reviewers performed the quality assessment separately and disagreements were resolved by consensus in the presence of a third reviewer. Overall, nine of the included articles were of good quality and 16 were of fair quality. No article was excluded on the basis of poor methodological quality, rather scores were used in considering the quality of the evidence. Results of the quality appraisal are detailed in Table 2.

**TABLE 2:** Results of risk of bias and quality assessment.

Axis tool items (Downes et al., 2016)	1	2	3	4	5	6	7	8	9	10	11	12	13	14	15	16	17	18	19	20	21	22	23	24	25
Aims/objectives of the study clear?	+	+	+	+	+	+	+	+	+	+	+	+	+	+	+	+	+	+	+	+	+	+	+	+	+
Study design appropriate for the stated aim(s)?	+	+	+	+	+	+	+	+	+	+	+	+	+	+	+	+	+	+	+	+	+	+	+	+	+
Sample size justified?	+	−	+	−	−	−	−	−	−	−	−	+	+	−	−	+	+	+	−	+	+	−	−	+	−
Target/reference population clearly defined?	+	+	+	+	+	+	+	+	+	+	+	+	+	+	+	+	+	+	+	+	−	+	+	+	+
Was the sample frame taken from an appropriate population base so that it closely represented the target population under investigation?	+	+	+	+	+	+	+	?	+	?	+	+	+	+	+	+	+	+	+	+	−	+	?	+	+
Was the selection process likely to select subjects/participants that were representative of the target/reference population under investigation?	+	+	+	−	−	+	−	+	−	−	−	−	−	+	+	−	−	−	+	+	−	−	−	+	+
Were measures undertaken to address and categorize non‐responders?	−	−	−	+	+	−	+	+	−	−	−	−	−	−	−	−	−	−	−	−	−	+	−	−	+
Were the risk factor and outcome variables measured appropriate to the aims of the study?	+	+	+	+	+	+	+	+	+	+	+	+	+	+	+	+	+	+	+	+	+	+	+	+	+
Were the risk factor and outcome variables measured correctly using instruments that had been trialled, piloted or published previously?	+	+	+	+	+	+	+	+	+	+	−	+	+	−	+	−	−	+	+	+	−	+	+	−	+
Is it clear what was used to determine statistical significance and/or precision estimates? (e.g. *p*‐values, confidence intervals	+	+	+	+	+	+	+	+	+	+	+	+	+	+	+	+	+	+	+	−	+	+	+	+	+
Were the methods sufficiently described to enable them to be repeated?	+	+	+	+	+	+	+	+	+	+	+	+	+	+	+	+	+	+	+	−	+	+	+	+	+
Were the basic data adequately described?	+	+	+	+	+	−	+	+	+	+	+	+	+	+	+	+	−	+	+	+	+	+	+	+	+
Does the response rate raise concerns about non‐response bias?	−	−	?	−	−	?	−	+	?	?	?	−	−	−	+	?	?	−	?	−	+	−	−	?	−
If appropriate, was information about non‐responders described?	+	−	−	+	+	−	+	+	+	−	−	+	−	−	−	−	−	+	−	−	−	+	−	−	+
Were the results internally consistent?	+	+	+	+	+	+	+	+	+	+	+	+	+	+	+	+	+	+	+	+	+	+	+	+	+
Were the results presented for all the analyses described in the methods?	+	+	+	+	+	+	+	+	+	+	+	+	+	+	+	+	+	+	+	+	+	+	+	+	+
Were the authors' discussions and conclusions justified by the results?	+	+	+	+	+	+	+	+	+	+	+	+	+	+	+	+	−	+	+	+	+	+	+	+	+
Were the limitations of the study discussed?	+	+	+	+	+	+	+	+	+	+	+	+	+	−	+	+	−	+	+	+	+	+	+	+	−
Were there any funding sources or conflicts of interest that may affect the authors' interpretation of the results?	−	−	−	−	−	−	−	−	−	−	−	−	−	−	−	−	−	−	−	−	−	−	−	−	−
Was ethics approval or consent of participants attained?	+	+	+	+	+	+	+	+	+	+	+	+	+	+	−	+	−	+	+	+	+	+	+	+	+
Total Quality Score	17	15	16	16	16	14	16	17	15	13	13	16	15	13	15	14	10	16	15	14	13	16	13	15	16

*Note*: + = Yes; − = No;? = Do not know.

1 = Avestan et al., 2015; 2 = Aboumatar et al., 2015; 3 = Amininasab et al., 2017; 4 = Liu et al., 2020; 5 = Philipp et al., 2016; 6 = Bagheri et al., 2018a, 2018b; 7 = Chochinov et al., 2002; 8 = Houmann et al., 2014; 9 = Bovero et al., 2021; 10 = Bagheri et al., 2018a; 11 = Ferri et al., 2015; 12 = Bagheri et al., 2018b; 13 = Di Lorenzo et al., 2018; 14 = Aydın Er et al., 2018; 15 = Julião et al., 2017; 16 = Karimi et al., 2019; 17 = Khorasanizadeh et al., 2018; 18 = Monforte‐Royo et al., 2018; 19 = Oechsle et al., 2014; 20 = Salehi et al., 2020; 21 = Tauber‐Gilmore, Norton, et al., 2018; 22 = Vehling & Mehnert, 2014; 23 = Wang et al., 2019; 24 = Zirak et al., 2017; 25 = Hack et al., 2004.

### 3.6 Data abstraction

Two reviewers independently extracted data from the included articles using the Covidence data extraction table that was modified to suit the purpose of the review. Differences were discussed between the two reviewers to reach a consensus, with no discrepancies requiring resolution by a third reviewer. Characteristics of the included studies are detailed in Table 3.

**TABLE 3:** Characteristics of studies.

Article	Aim	Design	Dignity PROM used	Sample size and diagnostic population	Setting and country	Mean age (SD) in years, gender, and sampling technique	Summary of results
Aboumatar et al., 2015	Test feasibility of quantitative assessment of patients' experiences with dignity and respectful care in the ICU	Cross‐sectional	Patient Dignity Inventory (PDI)	30 patients and 41 family members	Intensive care unit (ICU), USA	Patients: 56 (16), Male: 52%. Family members: 56 (14), Male: 32% Convenience sampling	Most reported source of loss of dignity was symptom distress and the least reported source of loss of dignity was social support. Amongst psychological variables, the highest sources of loss of dignity were anxiety and depression.
Amininasab et al., 2017	Determine the relationship between human dignity and medication adherence in patients with heart failure	Cross‐sectional.	PDI‐Persian version	300 patients with heart failure	Cardiac hospital, Iran	64.15, Male: 50% Census sampling	Severe loss of dignity reported. Loss of dignity associated with medication adherence (*r* = −.6, *p* < .001)
Avestan et al., 2015	Explore cancer patient perceptions of respecting their dignity and related variables in an Iranian cancer specific centre	Descriptive correlational	PDI	250 patients with cancer	Cancer specific Hospital, Iran	50.5 (17.7), Male: 50% Convenience sampling	Moderate to severe loss of dignity reported. Loss of dignity associated with age (r = 0.14, *p* = .027), being illiterate (*p* = .01), unemployment, (*p* = .001), less income (*p* = .001), history of disease recurrence (*p* = .03)
Aydın Er et al., 2018	Explore opinions and experiences of Turkish patients and nurses about respectful care of human dignity	Descriptive cross‐sectional	Self‐developed dignified care questionnaire	150 patients with cardiac, neurological, and neurosurgical conditions	Cardiology, neurology and neurosurgery units, Turkey	59.4 (16.6), Male: 48.7% Sampling type not specified	Majority (80.0%) were satisfied with dignified care. Most important elements for dignified care: nurses' protection of patients' rights (86.0%), good communication and respect for privacy (84.7%). Differences in views about dignified care: age (*p* = .035), duration of illness (*p* = .038), and frequency of hospitalization (*p* ≤ .030).
Bagheri et al., 2018a; Bagheri et al., 2018b	Investigate factors related to dignity in patients with heart failure and test the validity of the Dignity Model	Descriptive‐correlational	Inherent Dignity Questionnaire (IDQ), Social Dignity Questionnaire (SDQ), Dignity Conserving Repertoire Questionnaire (DCRQ)	130 inpatients with heart failure	Cardiac wards in urban hospitals, Iran	62 (13.9), Male: 67.7% Convenience sampling	Dignity correlated with frequency of hospitalization (*p* = .040), illness‐related worries (*p* = .001), social dignity (*p* = .032), and dignity conserving repertoire (*p* < .001). Illness‐related worries correlated: dignity conserving repertoire score, and social dignity.
Bovero, Sedghi, Botto, et al., 2018	Investigate dignity amongst end‐of‐life cancer patients, and assess the relationship between dignity and other patients' psychosocial and spiritual variables	Cross‐sectional	PDI‐Italian Version	127 inpatients with cancer	Cancer hospice hospital, Italy	75.2 (11.40), Male: 51.2% Sampling type not specified	Loss of dignity correlated with anxiety (*p* = .000), depression (*p* = .000), quality of life (*p* = .000), and demoralization (*p* = .000), physical well‐being (*p* = .000), emotional well‐being (*p* = .000), functional well‐being (*p* = .000), hope (*p* = .002), and coping style: denial (*p* = .000) and self‐blame (*p* = .000). Predictors of dignity: emotional well‐being (*p* < .01), self‐blame (*p* < .01), Depressive symptomatology (*p* < .01), physical well‐being (*p* < .05).
Bovero et al., 2021	Investigate the relationship between personality traits and dignity in cancer patients nearing death	Cross‐sectional	PDI‐Italian version	210 patients with End‐of‐life cancer Inpatients = 147 (70%), Hospice = 63 (30%)	Palliative Care Unit or ward, Italy	67.83 (11.61), Male: 55.7% Sampling type not specified	Significant association between dignity and personality traits including Agreeableness (*p* ≤ .05), and Neuroticism (*p* ≤ .01). Personality traits significantly associated with PDI subscales: Conscientiousness associated with Social Support; Agreeableness with Existential Distress and Loss of Purpose and Meaning; Neuroticism with Psychological Distress, Social Support, and Physical Symptoms; Dependency with Existential Distress and Loss of Purpose and Meaning
Bovero, Sedghi, Opezzo, et al., 2018	Assess the prevalence of the dignity‐related existential distress in patients with end‐of‐life cancer	Cross‐sectional	PDI‐Italian Version	207 inpatients with cancer	Palliative care unit, Italy	67.95 (14.15), Males: 48.8% Sampling type not specified	18.8% of patients identified dignity‐related existential distress. Dignity‐related existential distress associated with demoralization (*p* < .001), age (*p* < .05), and coping styles: “self‐blame” (*p* < .001) and “positive reframing” (*p* < .05).
Chochinov et al., 2002	Identify dying patients perception of their ability to maintain a sense of dignity	Cross‐sectional, cohort study	7‐point sense of dignity item	213 patients with cancer, Inpatients = 170 (80%), Outpatients = 43 (20%)	Palliative care unit or ward, Canada	69 (12·6), Male: 95 (45%) Sampling type not specified	Strong sense of dignity reported: 54% High dignity levels associated with optimal level of independence.
Di Lorenzo et al., 2018	Validation of the PDI amongst patients hospitalized in an acute psychiatric ward	Validation study	PDI‐Italian Version	165 inpatients with acute mental health illness	Psychiatric ward, Italy	43.89 (14.42), Male:45% Sampling type not specified	Mild loss of dignity reported. Loss of dignity correlated with insufficient economic status (*p* = .002), suicidal risk (*p* = .004).
Ferri et al., 2015	Explore inpatients' perception of dignity	Descriptive cross‐sectional	Self‐developed dignified care questionnaire	100 inpatients of medical‐surgical wards.	10 Medical‐surgical wards of a General Hospital, Italy	Means and SD not specified. Male: 48% Purposive sampling	Contributors to dignity: Respectful nurse–patient interaction (69%), maintenance of physical privacy (68.6%), information and autonomy (59%).
Hack et al., 2004	Analyse dignity data gathered from cancer patients having less than 6 months to live	Cross‐sectional study	7‐point sense of dignity item	213 patients with terminal cancer Inpatients = 170 (80%), Outpatients = 43 (20%)	Palliative care, Canada	69:0 (12:6), Male: 95 (45%) Consecutive sampling	High sense of dignity: 54% Significant loss of dignity: 7.5% Sense of dignity correlated with: Quality of Life, Depression, and Intimate Dependency (*p* < .001)
Houmann et al., 2014	Investigate participation in, and evaluation of, Dignity Therapy (DT) and longitudinal changes in patient‐rated outcomes	Prospective (pre/post)	DT Question Framework (DTQF), PDI	80 patients with cancer: Inpatients = 48 (60%), Outpatients = 12 (15%), Home‐care patients = 20 (25%)	Palliative care unit, Denmark	63 (13), Male: 40% Consecutive sampling	DT: helpful (73%), satisfactory (89%), effective in improving depression, anxiety, sense of dignity, and hopelessness.
Julião et al., 2017	Determine the influence of DT on demoralization syndrome, the desire for death, and a sense of dignity in terminally ill inpatients	Nonblinded phase II randomized controlled trial	PDI	80 inpatients with palliative care needs: Intervention (DT) (41 patients) and standard palliative care Control (39 patients)	Palliative care unit of a tertiary hospital, Portugal	Control group: 66.1 (12.9), Male: 43.9% DT group: 20 (51.3), Male: 48.7% Random sampling	DT was effective in reducing loss of dignity, demoralization syndrome, and desire for death
Karimi et al., 2019	Investigate the perceptions of hospitalized older adults about the importance and observance of dignity	Cross‐sectional	Self‐developed dignity importance and observance questionnaire.	400 hospitalized older patients (≥ 60), 146 Nurses	Medical‐surgical Hospital, Iran	69.53, Males: 43% Convenience sampling	Good to very good observance of dignity: 70.3% High levels of dignity in ICU and Ear, Nose and Throat (ENT) wards. Moderate levels of dignity in the emergency department and surgical wards. Low levels of dignity in emergency surgical and female surgical wards. Observance of dignity associated with economic status, gender, marital status, type of ward, bed layout, and length of hospitalization
Khorasanizadeh et al., 2018	Compare nurses, nursing students, and patients' attitudes about the observance of the patient's dignity	Cross‐sectional	Self‐developed patient's dignity observance questionnaire	100 inpatients with mental health illness, 100 Nurses, 100 Nursing students	Psychiatric unit of a hospital, Iran	Mean and SD not specified, Males:46% Purposive sampling	Observance of dignity was higher in Kashan City compared with Tehran City
Liu et al., 2020	Evaluate the effects of meaning in life and individual characteristics on dignity in patients with advanced cancer	Cross‐sectional	PDI	167 patients with advanced cancer, Inpatients = 88 (53%), Home patients = 79 (47%)	Palliative care unit, China	60.3 (11.7), Male: (52%) Sampling type not specified	Mild loss of dignity was reported in 78.4% of patients. Moderate to severe loss of dignity reported in 22% of patients. Loss of dignity associated with meaning in life (*p* < .01)
Monforte‐Royo et al., 2018	Test a model of perceived loss of dignity, symptoms of depression and functional impairment, as risk factors for wish to hasten death in advanced cancer patients	Cross‐sectional	PDI‐Spanish version	193 inpatients with cancer	Oncology unit, Spain	62.6 (SD 9.9), Males: 58.5% Sampling type not specified	Mild loss of dignity reported. Loss of dignity associated with functional impairment, perceived loss of control, and wish to hasten death,
Oechsle et al., 2014	Evaluate the impact of symptom burden, distress, overall condition, and individual patient characteristics on self‐perceived dignity in terminally ill cancer patients	Cross‐sectional	PDI‐German version	61 inpatients with cancer	Inpatient palliative care ward, Germany	64 years (36–85), Males: 41% Consecutive sampling	Moderate loss of dignity reported. Loss of dignity correlated with overall psychological distress (*p* < .001), lack of energy (*p* < .001), anxiety (*p* < .001), sadness (*p* = .002), pain (*p* = .009), shortness of breath (*p* = .019), irritability (*p* = .023), thirst (*p* = .029), and tiredness (*p* = .041).
Philipp et al., 2016	Determine the extent to which cancer patients experience loss of dignity during primary cancer care (baseline) and at 3‐month follow‐up and the contribution of positive social support and detrimental social interactions on loss of dignity at follow‐up	Prospective longitudinal study	Sense of dignity item	270 patients with cancer: Inpatients = 146 (54%), Outpatients = 124 (46%)	Oncology and haematology units, Germany	56.9 (13.9), Males: (53.3%) Sampling type not specified	Severe loss of dignity reported. Loss of dignity associated with physical problems (*p* = .001), depression (*p* = .003) and detrimental interactions (*p* = .02).
Salehi et al., 2020	Investigate the relationship of respect for dignity with anxiety, depression, stress and quality of life in patients with heart failure	Cross‐sectional	Inherent Dignity questionnaire (IDQ)	150 inpatients with heart failure	Cardiology unit, Iran	58.72 (12.53), Male: 56.7% Purposive sampling	Moderate level of dignity reported. Dignity correlated with quality of life (*p* = .002), depression (*p* = .004), anxiety (*p* = .001) and stress (*p* = .001), marital status (*p* = .04) employment status (*p* = .03).
Tauber‐Gilmore, Norton, et al., 2018; Tauber‐Gilmore, Addis, et al., 2018	Develop tools for measuring patient dignity in acute hospitals	Mixed‐methods interventional validation study	Patient Dignity Survey Tool.	3611 pre‐intervention inpatients and 2082 post intervention inpatients	Medical‐surgical, oncology and older patient unit, England	Not reported	Communication training for HCPs had a positive impact on patients' dignity: Mean dignity score increased after intervention (*p* < .001).
Vehling & Mehnert, 2014	Test the hypothesis that loss of dignity mediates the association between the number of physical problems and demoralization in a sample of cancer patients	Cross‐sectional	PDI	112 inpatients with cancer	Oncological wards and community medical clinic, Germany	56.0 (14.1), Males: 57% Consecutive sampling	Mild loss of dignity reported. Loss of dignity associated with gender (*p* = .014), depression (*p* < .001), physical problems (*p* < .001), and demoralization (*p* < .001).
Wang et al., 2019	Examine loss of dignity for patients with early and advanced cancer in Mainland China	Cross‐sectional survey	PDI	202 inpatients with cancer	Tumour hospital, China	≤44 Years: 14.9%, 45–59 years: 44.1%, ≥60 years: 41.1% Sampling type not specified	Mild loss of dignity reported in 71% of patients. Moderate to severe loss of dignity reported in 23% of patients. Loss of dignity correlated with age (*p* = .009), cancer stage (*p* = .001), psychological distress (*p* = <.001), anxiety (*p* = <.001), depression (*p* = <.001), and Symptom burden (pain, fatigue, nausea, disturbed sleep, feeling upset, shortness of breath, loss of appetite, drowsiness, dry mouth, vomiting, sadness, numbness, activity, mood, working, relationship with people, walking, enjoyment of life) (*p* = <.001)
Zirak et al., 2017	Assess the extent to which patients' dignity is respected and evaluate its relationship with contextual variables	Cross‐sectional	Self‐developed patient's dignity preservation questionnaire	256 patients	Not specified, Iran	42.64 (20), Males:46% Cluster sampling	Moderate levels of dignity reported. Levels of dignity associated with marital status (*p* = .012), living in city or village (*p* = .013), and type of hospital (*p* = .004).

### 3.7 Data synthesis

The quantitative synthesis approach proposed by Schick‐Makaroff et al. (2016) was used to summarize, synthesize and integrate the extracted data. Whilst the initial intention was to perform a meta‐analysis, this was not possible because the included studies lacked uniformity in outcome measures. Nonetheless, results from the studies included in the review were first summarized into tables in terms of their methodological quality (Table 2), and general characteristics including a summary of findings (Table 3). The summarized data were then synthesized and integrated to produce summary statements. The key findings are presented in six areas: (i) Description of the included studies. (ii) Prevalence of loss of dignity. (iii) Facilitators of dignity or dignified care. (iv) Association between dignity and demographic variables. (v) Association between dignity and clinical variables. (vi) Association between dignity and psychosocial variables.

## 4 RESULTS

### 4.1 Description of the included studies

Multiple studies were undertaken in Iran (*n* = 7) (Amininasab et al., 2017; Avestan et al., 2015; Bagheri et al., 2018a; Karimi et al., 2019; Khorasanizadeh et al., 2018; Salehi et al., 2020; Zirak et al., 2017), Italy (*n* = 5) (Bovero et al., 2021; Bovero, Sedghi, Botto, et al., 2018; Bovero, Sedghi, Opezzo, et al., 2018; Di Lorenzo et al., 2018; Ferri et al., 2015), Germany (*n* = 3) (Oechsle et al., 2014; Philipp et al., 2016; Vehling & Mehnert, 2014), China (*n* = 2) (Liu et al., 2020; Wang et al., 2019) and Canada (*n* = 2) (Chochinov et al., 2002; Hack et al., 2004). Single studies were conducted in Turkey (Aydın Er et al., 2018), Spain (Monforte‐Royo et al., 2018), England (Tauber‐Gilmore, Norton, et al., 2018), Portugal (Julião et al., 2017), USA (Aboumatar et al., 2015) and Denmark (Houmann et al., 2014). Characteristics of the included studies are presented in Table 3.

The majority of studies (*n* = 20) employed cross‐sectional designs. Two papers reported validation studies (Di Lorenzo et al., 2018; Tauber‐Gilmore, Norton, et al., 2018). Also included were a randomized controlled trial (RCT) (Julião et al., 2017), a pre‐test‐post‐test (Houmann et al., 2014), and a longitudinal study (Philipp et al., 2016).

Data collection instruments used to measure dignity included the Patient Dignity Inventory (PDI) (*n* = 14) (Chochinov et al., 2008), the Inherent Dignity Questionnaire (*n* = 2) (Bagheri et al., 2014), the Patient Dignity Survey Tool (*n* = 1) (Tauber‐Gilmore, Norton, et al., 2018) and the Sense of Dignity Item (*n* = 3). Five studies (Aydın Er et al., 2018; Ferri et al., 2015; Karimi et al., 2019; Khorasanizadeh et al., 2018; Zirak et al., 2017) utilized study specific unvalidated instruments.

The majority of studies (*n* = 21) only included patients. Three studies (Aydın Er et al., 2018; Karimi et al., 2019; Khorasanizadeh et al., 2018) included patients and HCPs, and one study (Aboumatar et al., 2015) recruited patients and family members. Studies included 71 (Aboumatar et al., 2015) to 5693 individual participants (Tauber‐Gilmore, Norton, et al., 2018). Mean ages ranged from 43.9 years (Di Lorenzo et al., 2018) to 75.2 years (Bovero, Sedghi, Botto, et al., 2018). One study (Karimi et al., 2019) exclusively included patients 60 years and older.

More than half (*n* = 13) of the studies included patients with cancer receiving care in acute oncology and/or palliative care settings. Three studies (Amininasab et al., 2017; Bagheri et al., 2018a; Salehi et al., 2020) involved patients with heart failure in cardiology units and two studies (Di Lorenzo et al., 2018; Khorasanizadeh et al., 2018) involved patients with mental health conditions in inpatient mental health units and one study (Aboumatar et al., 2015) involved patients (and their families) in an intensive care unit (ICU). The remaining four studies recruited patients with varied conditions in medical and surgical units (Aydın Er et al., 2018; Ferri et al., 2015; Karimi et al., 2019; Tauber‐Gilmore, Norton, et al., 2018).

### 4.2 Prevalence of loss of dignity

More than half of the studies (*n* = 15) provided an operational definition of the construct of dignity and/or dignified care as part of the background of the study. Most studies assessed levels of dignity using the PDI and the 7‐point Sense of Dignity Item, a 1‐item question on a 7‐point Likert‐type scale (0 = no sense of lost dignity/sense of dignity is strong, 1 = minimum loss of sense of dignity, 2 = mild loss of sense of dignity, 3 = moderate loss of sense of dignity, 4 = strong loss of sense of dignity, 5 = severe loss of sense of dignity and 6 = extreme loss of sense of dignity) (Chochinov et al., 2002; Hack et al., 2004). Analysis of the data revealed that studies conducted in Europe and Canada reported a relatively low prevalence of loss of dignity as compared with studies conducted in Asian countries. For instance, two studies in Canada that measured levels of dignity using the 7‐point sense of dignity item (Chochinov et al., 2002; Hack et al., 2004) observed that more than half (54%) of patients reported a strong sense of dignity, with only 7.5% reporting a moderate to severe loss of dignity. Similarly, three studies from Europe (Di Lorenzo et al., 2018; Monforte‐Royo et al., 2018; Vehling & Mehnert, 2014) presented evidence of a mild loss of dignity, with two studies from Germany reporting moderate (Oechsle et al., 2014) to severe (Philipp et al., 2016) loss of dignity. In contrast, the majority of studies conducted in Asia included evidence of severe loss of dignity (Amininasab et al., 2017; Avestan et al., 2015;Liu et al., 2020; Wang et al., 2019). Two studies from China (Liu et al., 2020; Wang et al., 2019) reported a moderate to severe loss of dignity in 22%–23% of patients. Similarly, three Iranian studies (Amininasab et al., 2017; Avestan et al., 2015; Salehi et al., 2020) recorded a moderate to severe loss of dignity amongst patients involved in the studies.

### 4.3 Facilitators of dignity or dignified care

Three studies (Aydın Er et al., 2018; Ferri et al., 2015; Karimi et al., 2019) identified factors that facilitated dignity or dignified care included nurses' protection of patients' rights, effective HCP‐patient communication and respecting privacy. Experimental studies in Portugal (Julião et al., 2017) and Denmark (Houmann et al., 2014) reported the positive impact of dignity therapy in promoting dignity. An interventional study in the UK (Tauber‐Gilmore, Norton, et al., 2018) identified that communication training for HCPs improved patients' dignity.

### 4.4 Association between dignity and demographic variables

Findings of studies on the association between dignity and demographic variables varied. Five studies (Avestan et al., 2015; Bagheri et al., 2018a; Bovero, Sedghi, Opezzo, et al., 2018;Chochinov et al., 2002; Liu et al., 2020) identified that younger age was associated with lower dignity levels. Alternatively, two studies (Bovero, Sedghi, Botto, et al., 2018; Philipp et al., 2016) found no significant association between dignity and age.

Four studies (Avestan et al., 2015; Di Lorenzo et al., 2018; Karimi et al., 2019; Salehi et al., 2020) established an association between economic or employment status and dignity, where lower dignity levels were found to be associated with unemployment or lower income status.

Two studies (Karimi et al., 2019; Vehling & Mehnert, 2014) identified a relationship between dignity and gender, where dignity was more important to women (Karimi et al., 2019), and loss of dignity was higher for women than men (Vehling & Mehnert, 2014). Conversely, five studies (Bagheri et al., 2018a; Bovero, Sedghi, Botto, et al., 2018; Chochinov et al., 2002; Liu et al., 2020; Philipp et al., 2016) did not establish an association between dignity and gender.

Three studies (Karimi et al., 2019;Salehi et al., 2020; Zirak et al., 2017) identified an association between dignity and marital status. In contrast, four studies (Bagheri et al., 2018a; Bovero, Sedghi, Botto, et al., 2018; Chochinov et al., 2002; Liu et al., 2020) did not find a correlation between dignity and marital status. The studies that observed differences in dignity based on marital status revealed dignified care was more important to individuals who were not married compared with those who were married (Karimi et al., 2019). In addition, individuals who were not married reported significantly lower levels of dignity compared with those who were married (Salehi et al., 2020; Zirak et al., 2017).

The relationship between dignity and levels of education also differed between studies. One study (Avestan et al., 2015) observed that individuals without a formal education reported a greater loss of dignity compared with those with formal education. In contrast, two studies (Bagheri et al., 2018a; Chochinov et al., 2002) did not find an association between dignity and educational status.

### 4.5 Association between dignity and clinical variables

Four studies (Bagheri et al., 2018a; Chochinov et al., 2002; Karimi et al., 2019; Liu et al., 2020) observed differences in dignity based on hospitalization/inpatient status or frequency of hospitalization. Two studies (Chochinov et al., 2002; Liu et al., 2020) that recruited samples from both inpatient and outpatient populations identified that lower dignity levels were associated with hospitalization or inpatient status. Individuals with fewer hospitalisations had higher dignity levels (Bagheri et al., 2018a). Levels of dignity also differed based on the type of hospital ward (Karimi et al., 2019) with higher levels of dignity reported for patients admitted to ICU and Ear, Nose and Throat (ENT) wards, moderate levels of dignity for patients admitted to the emergency department, infectious diseases and surgical wards and low levels of dignity for those in emergency surgical and female surgical wards. Characteristics of these wards were not described in the article. Further, individuals who were hospitalized in beds that offered greater privacy (private rooms, spaces) were found to have higher levels of dignity compared with those in shared rooms (Karimi et al., 2019). One study (Avestan et al., 2015) reported differences in dignity based on disease recurrence with lower dignity levels in patients with a history of disease recurrence compared with those without a history of disease recurrence.

Three studies (Chochinov et al., 2002; Hack et al., 2004; Monforte‐Royo et al., 2018) reported an association between dignity loss and functional impairment. Symptoms such as fatigue, pain, shortness of breath, anxiety, sadness, irritability and tiredness were also identified as predictors of loss of dignity (Liu et al., 2020; Oechsle et al., 2014; Philipp et al., 2016; Wang et al., 2019). Two studies (Liu et al., 2020; Wang et al., 2019) reported a correlation between dignity and stage of cancer; however, two further studies (Bovero, Sedghi, Botto, et al., 2018; Philipp et al., 2016) did not identify this correlation. Five studies (Bovero, Sedghi, Botto, et al., 2018; Chochinov et al., 2002; Hack et al., 2004; Salehi et al., 2020; Wang et al., 2019) established an association between the quality of life and dignity with a higher quality of life associated with higher dignity levels. One study (Amininasab et al., 2017) found a negative correlation of adherence to medication with loss of dignity.

### 4.6 Association between dignity and psychosocial variables

A positive correlation of overall psychological distress with loss of dignity was reported in three studies (Bagheri et al., 2018a; Oechsle et al., 2014; Wang et al., 2019), with a reduction in psychological distress associated with an improvement in dignity. Several studies (*n* = 6) identified or found a relationship between depression and loss of dignity (Bovero, Sedghi, Botto, et al., 2018; Chochinov et al., 2002; Hack et al., 2004; Philipp et al., 2016; Salehi et al., 2020; Vehling & Mehnert, 2014; Wang et al., 2019). One study (Monforte‐Royo et al., 2018) observed a relationship between dignity, depression and a wish to hasten death, with depression co‐occurring with a wish to hasten death to predict loss of dignity. High levels of anxiety were found to be associated with dignity loss in three of the included studies (Chochinov et al., 2002; Salehi et al., 2020; Wang et al., 2019).

Several studies reported differences in levels of dignity based on coping strategies (Bovero, Sedghi, Botto, et al., 2018; Bovero, Sedghi, Opezzo, et al., 2018). Negative coping strategies, such as self‐blame, emerged as a predictor of loss of dignity (Bovero, Sedghi, Botto, et al., 2018), whilst positive coping strategies (e.g. active coping, positive reframing and self‐distraction) were associated with higher levels of dignity (Bovero, Sedghi, Opezzo, et al., 2018).

Three studies (Bovero, Sedghi, Botto, et al., 2018; Bovero, Sedghi, Opezzo, et al., 2018; Vehling & Mehnert, 2014) found a positive correlation between demoralization and loss of dignity. Other psychological variables observed to be associated with dignity included meaning of life (Liu et al., 2020), spirituality (Bovero, Sedghi, Botto, et al., 2018), detrimental interactions with close relatives (Philipp et al., 2016), suicidal risk (Di Lorenzo et al., 2018) and personality traits (Bovero et al., 2021).

## 5 DISCUSSION

Our review findings identified patients' dignity as a dynamic concept influenced by combinations of demographic, clinical and psychological factors. Levels of dignity amongst patients were variable across countries, and in some cases disturbing, and this may be attributed to differences in healthcare systems across regional areas (i.e. Asia and Europe) (Ahmed et al., 2019; Asandului et al., 2014). Our findings also revealed that patients' dignity is preserved when HCPs communicate effectively with them, protect their privacy and provide them with dignity therapy, a finding consistent with previous research (Clancy et al., 2021; Lin et al., 2013; Xiao et al., 2019). The above discourse highlights the universal relevance of effective HCP‐patient communication, patient privacy and dignity therapy to patients' dignity and calls on the need for HCPs to imbibe them with care to promote patients' dignity and/or dignified care.

The majority of studies included in the review provided an operational definition of the construct of dignity and these definitions aligned with the concept of extrinsic or social dignity that has been described elsewhere in the literature (Allard et al., 2018; Jacobson, 2007, 2009). Studies included in the current review employed different dignity‐related instruments with different scoring criteria for classifying levels of dignity. Further, amongst studies that measured levels of dignity using the same data collection instrument (i.e. PDI) (Amininasab et al., 2017; Avestan et al., 2015; Liu et al., 2020; Wang et al., 2019), different scoring criteria were employed to classify the loss of dignity. Future studies involving large samples from different countries but using the same instruments and scoring criteria will provide more information on the differences in levels of the dignity of patients based on geographical locations.

The current review identified the PDI as the most widely used dignity‐related instrument accounting for more than half of studies, with most research on patient dignity focused on patients with cancer, highlighting a gap in understanding of differences between care settings. Previous research has attributed the dominance of the PDI to its availability in several languages which makes it readily accessible and available for HCPs and researchers across countries (Bagnasco et al., 2020).

The present review identified several factors that promote patients' dignity during acute hospitalization including effective HCP‐patient communication, communication skills training for HCPs, and patient privacy. These factors have been highlighted in previous reviews as important elements for patient dignity (Clancy et al., 2021; Lin et al., 2013). Also similar to the findings of previous research, the current review identified dignity therapy as an important intervention for promoting patients' dignity (Martínez et al., 2017; Xiao et al., 2019). Dignity therapy is a brief, individualized psychotherapy aimed at promoting dignity and reducing distress amongst patients whose lives are threatened by illness (Martínez et al., 2017). Dignity therapy offers patients an opportunity to discuss issues that matter most to them, with sessions recorded, transcribed, edited and with a final version presented back to the patient. When satisfied with it, the patient could bequeath it to a friend or family member (Chochinov et al., 2005; Hall et al., 2009). Health services might consider adopting dignity therapy into routine care. However, to enable this, HCPs will need resources as well as training to gain relevant knowledge and skills to effectively provide dignity therapy for patients and their families. In addition, communication skills training for HCPs may promote dignity for hospitalized patients, as undignified care has been linked to poor communication by HCPs (Beckstrand et al., 2012).

Our review identified an association between dignity and several patient demographic variables, lending credence to existing propositions in the literature that the dignity of a person is determined by their social status and influenced by their interaction with others in society (Jacobson, 2007; Nordenfelt, 2004). For instance, our review identified that lower dignity levels were associated with a lack of formal education and, or low economic status, a finding consistent with previous research in long‐term care settings (Dong et al., 2021; Kisvetrová et al., 2021). Our review also observed a relationship between dignity and marital status, previously identified amongst the end of life patients (Albers et al., 2013). Further, our review identified that younger patients were more vulnerable to loss of dignity compared with older patients. This finding is, however, contrary to previous research that has cited advancing age as a risk factor for loss of dignity due to declining physical and cognitive functions associated with ageing (Filipska et al., 2020; Hubbard et al., 2017; Tracy & Skillings, 2007). These findings highlight that HCPs need to be aware of the varying influence of sociodemographic variables on patients' dignity for timely intervention.

Amongst the studies included in the current review that recruited samples from inpatient and outpatient populations, hospitalization was associated with lower dignity levels, and fewer prior hospitalizations were associated with an improvement in levels of dignity. This finding is in tandem with existing evidence that hospitalization poses a significant threat to the physical and psychosocial well‐being of patients (Hillman et al., 2013; Stewart & Arora, 2018). Hospitalized patients are at risk of experiencing physical and emotional abuse that can undermine their overall well‐being, including their dignity (Filipska et al., 2020; Naderi et al., 2019). Levels of dignity also differed based on the type of hospital ward, with the highest dignity in ICU and ENT wards and lowest dignity in emergency surgical and female surgical wards. Delay in response to patients' needs, increased frequency of treatment errors, exposure to violence and overcrowding, are some of the experiences of patients in emergency departments (Morley et al., 2018), that have the tendency to undermine dignity (Asmaningrum et al., 2020).

Another key finding of the current review was the association between dignity and individual patient characteristics. Functional impairment, physical symptoms and stage of cancer were predictors for loss of dignity. Previous research has similarly identified the influence of poor health status, physically distressing symptoms and impaired functional ability as determinants of loss of dignity (Albers et al., 2013; Nouri et al., 2017). In addition, the current review observed a positive correlation between the quality of life and dignity, which supports the earlier work by George (1998) who postulated that a strong sense of dignity, together with a strong social identity and a sense of control, were sufficient for a good quality of life even when other determinants were unfavourable. The relationship between dignity and patients' characteristics is further espoused by Henry et al.'s (2015) conceptual model of dignity for patients in the ICU. Henry et al. (2015) identified three sources of patients' dignity (i.e., shared humanity, personal narratives, autonomy), each of which warrants respect. The personal narrative was defined as a type of dignity associated with an individual's achievements or social status, which is influenced by alterations imposed by sickness and or hospitalization.

Several studies included in this review identified depression, anxiety, demoralization, negative coping mechanism and overall psychological distress as predictors of loss of dignity. Consistent with our finding, depression and anxiety have been cited in previous studies as predictors of loss of dignity in outpatients (Grassi et al., 2017; Kostopoulou et al., 2018), and inpatients in long‐term care settings (Kisvetrová et al., 2021; Solomon et al., 2016). Loss of dignity has also been associated with demoralization and dysfunctional coping mechanisms such as hopelessness and helplessness in previous research (Grassi et al., 2017). Studies in the current review reported a relationship between dignity and spirituality or religiosity, and this concurs with the findings of previous studies that mentioned religion as an important determinant of dignity in end‐of‐life patients (Albers et al., 2013). However, our review finding is incongruent with the finding of a recent study that did not observe any association between dignity and spirituality amongst patients in long‐term care (Kisvetrová et al., 2021). Differences in healthcare needs between patients in acute care and those in long‐term care settings may account for the differences in findings. The provision of dignified care must, therefore, not only focus on action‐oriented responsibilities, but also, include measures for addressing patients' psychological well‐being since dignity is associated with patients' psychological health.

### 5.1 Limitations

A limitation of this review relates to the lack of homogeneity in the data presented in the included studies, which made it impossible for meta‐analysis to be performed. Second, studies included in the review measured levels of dignity and their association with related factors using several different data collection instruments with different scoring criteria, making data extraction and synthesis challenging as some of the studies did not provide all quantitative information.

### 5.2 Implications for practice

We suggest that, for patients' dignity to be promoted, the provision of care must include early detection of the perception of lowered dignity and measures aimed at alleviating patient discomfort, fostering functional independence and addressing psychosocial needs must be incorporated in care. Many papers were of low quality suggesting that more robust and high‐quality research on patient‐reported dignity and/or dignified care is needed.

## 6 CONCLUSION

The current systematic review represents a synthesis of quantitative evidence on levels of dignity in acute care settings and their association with demographic, clinical and psychological variables. Levels of dignity for hospitalized patients vary widely across geographical locations. Patients' dignity is upheld when HCPs communicate effectively, maintain their privacy and provide them with dignity therapy. Levels of dignity are influenced by demographic variables including age, marital status, gender, employment status and educational status. Dignity can also be influenced by clinical (i.e. hospitalization, type of hospital ward, functional impairment, physical symptoms, quality of life and stage of cancer) and psychological (i.e. depression, anxiety, demoralization and coping mechanisms) variables.

## AUTHOR CONTRIBUTIONS

**Table:** 

Criteria	Author Initials
Made substantial contributions to conception, design, extraction of data, data analysis and drafting of the manuscript	AGF
Involved in conceptualisation, data extraction and drafting of the manuscript. Also involved in reviewing the manuscript with insightful inputs.	DK
Participated in conceptualisation and reviewing the manuscript with insightful inputs.	LL
Participated in conceptualisation and reviewing the manuscript with insightful inputs.	HR
Participated in reviewing the manuscript with insightful inputs.	BR

## FUNDING INFORMATION

Deakin University.

## CONFLICT OF INTEREST

The research was supported by the Deakin University Postgraduate Research Office through the award of PhD scholarship to the first author (AGF).

### PEER REVIEW

The peer review history for this article is available at https://publons.com/publon/10.1111/jan.15370.

## Supporting information


Appendix S1

Appendix S2

## Data Availability

The data that support the findings of this review are available on request from the corresponding author.
