# Supplementary material for: A systematic review of patient‐reported dignity and dignified care during acute hospital admission
Source: J Adv Nurs. 2022 Jul 16;78(11):3540–58. doi: 10.1111/jan.15370 (PMC9795980; doi:10.1111/jan.15370)
Supplement: Supplementary file 2 — Appendix S2 [file JAN-78-3540-s001.docx]

**Supplementary file 2: Search strategy**

**MEDLINE Complete (via EBSCOhost platform)**

S33 S30 AND S31 AND S32

S32 S19 OR S20 OR S21 OR S22 OR S23 OR S24 OR S25 OR S26 OR S27 OR S28 OR S29

S31 S8 OR S9 OR S10 OR S11 OR S12 OR S13 OR S14 OR S15 OR S16 OR S17 OR S18

S30 S1 OR S2 OR S3 OR S4 OR S5 OR S6 OR S7

S29 (MH "Patient Outcome Assessment+") OR (MH "Outcome Assessment, Health Care+") OR (MH "Self Report") OR (MH "Patient Satisfaction+")

S28 TI “Cross Sectional*” OR AB “Cross Sectional*”

S27 TI Survey* OR AB Survey*

S26 TI Quantitative* OR AB Quantitative*

S25 TI Measure* OR AB Measure*

S24 TI Questionnaire* OR AB Questionnaire*

S23 TI Instrument* OR AB Instrument*

S22 TI Scale* OR AB Scale*

S21 TI Tool* OR AB Tool*

S20 TI "Patient reported experience measure*" ORAB "Patient reported experience measure*"

S19 TI "Patient reported outcome measure*" OR AB "Patient reported outcome measure*"

S18 (MH "Episode of Care") OR (MH "Terminal Care+") OR (MH "Palliative Care") OR (MH "Preoperative Care+") OR (MH "Post operative Care")

S17 TI Palliativ* OR AB Palliativ*

S16 TI Postoperative OR AB Postoperative

S15 TI Preoperative OR AB Preoperative

S14 TI oncolog* OR AB oncolog*

S13 TI “clinical setting*” OR AB “clinical setting*”

S12 TI Ward* OR AB Ward*

S11 TI Inpatient* OR AB Inpatient*

S10 TI “Acute Admission*” OR AB “Acute Admission*”

S9 TI Hospital* OR AB Hospital*

S8 TI “Acute Car*” OR AB “Acute Car*”

S7 (MH "Patient Reported Outcome Measures+")

S6 TI "car* in dignity" OR AB" car* in dignity"

S5 TI “Patient Reported Dignity” OR AB “Patient Reported Dignity”

S4 TI “Patient Dignity” OR AB “Patient Dignity”

S3 TI "dignity with car*" OR AB "dignity with car*"

S2 TI “car* with dignity” OR AB “car* with dignity”

S1 TI “dignified car*” OR AB “dignified car*”

**CINAHL (via EBSCOhost platform)**

S33 S30 AND S31 AND S32

S32 S19 OR S20 OR S21 OR S22 OR S23 OR S24 OR S25 OR S26 OR S27 OR S28 OR S29

S31 S8 OR S9 OR S10 OR S11 OR S12 OR S13 OR S14 OR S15 OR S16 OR S17 OR S18

S30 S1 OR S2 OR S3 OR S4 OR S5 OR S6 OR S7

S29 (MH "Patient-Reported Outcomes+") OR (MH "Outcomes (HealthCare)+") OR (MH "Nursing Outcomes")

S28 TI “Cross-Sectional*” OR AB “Cross-Sectional*”

S27 TI Survey OR AB Survey

S26 TI Quantitative* OR AB Quantitative*

S25 TI Measure* OR AB Measure*

S24 TI Questionnaire* OR AB Questionnaire*

S23 TI Instrument* OR AB Instrument*

S22 TI Scale OR AB Scale*

S21 TI Tool* OR AB Tool*

S20 TI “Patient Reported Experience Measure*” ORAB “Patient Reported Experience Measure*”

S19 TI "Patient reported outcome measure*" ORAB "Patient reported outcome measure*"

S18 (MH "Acute Care") OR (MH "Cardiac Care: Acute (Iowa NIC)") OR (MH "Post Anesthesia Care Units")

S17 TI Palliativ* OR AB Palliativ*

S16 TI Postoperative OR AB Postoperative

S15 TI Preoperative OR AB Preoperative

S14 TI oncolog* OR AB oncolog*

S13 TI “clinical setting*” OR AB “clinical setting*”

S12 TI Ward* OR AB Ward*

S11 TI Inpatient* OR AB Inpatient*

S10 TI “Acute Admission*” OR AB “Acute Admission*”

S9 TI Hospital* OR AB Hospital*

S8 TI “acute Car*” OR AB “acute Car*”

S7 (MH "Privacy and Confidentiality+") OR (MH"Human Dignity") OR (MH "Decision Making, Patient+") OR (MH "Patient Preference")

S6 TI "car* in dignity" OR AB "car* in dignity"

S5 TI “Patient Reported Dignity” OR AB “Patient Reported Dignity”

S4 TI “Patient Dignity” OR AB “Patient Dignity”

S3 TI "dignity with car*" OR AB "dignity with car*"

S2 TI “car* with dignity” OR AB “car* with dignity”

S1 TI “dignified car*” OR AB “dignified car*”

**AgeLine (via EBSCOhost platform)**

S32 S29 AND S30 AND S31

S31 S19 OR S20 OR S21 OR S22 OR S23 OR S24 OR S25 OR S26 OR S27 OR S28

S30 S8 OR S9 OR S10 OR S11 OR S12 OR S13 OR S14 OR S15 OR S16 OR S17 OR S18

S29 S1 OR S2 OR S3 OR S4 OR S5 OR S6 OR S7

S28 TI “Cross Sectional*” OR AB “Cross Sectional*”

S27 TI Survey* OR AB Survey*

S26 TI Quantitative* OR AB Quantitative*

S25 TI Measure* OR AB Measure*

S24 TI Questionnaire* OR AB Questionnaire*

S23 TI Instrument* OR AB Instrument*

S22 TI Scale* OR AB Scale*

S21 TI Tool* OR AB Tool*

S20 TI "Patient reported experience measure*" OR AB "Patient reported experience measure*"

S19 TI "Patient reported outcome measure*" OR AB "Patient reported outcome measure*"

S18 DE "Acute Care"

S17 TI Palliativ* OR AB Palliativ*

S16 TI Postoperative OR AB Postoperative

S15 TI Preoperative OR AB Preoperative

S14 TI oncolog* OR AB oncolog*

S13 TI “clinical setting*” OR AB “clinical setting*”

S12 TI Ward* OR AB Ward*

S11 TI Inpatient* OR AB Inpatient*

S10 TI “Acute Admission*” OR AB “Acute Admission*”

S9 TI Hospital* OR AB Hospital*

S8 TI “Acute Car*” OR AB “Acute Car*”

S7 DE "Dignity"

S6 TI "car* in dignity" OR AB "car* in dignity"

S5 TI “Patient Reported Dignity” OR AB “Patient Reported Dignity”

S4 TI “Patient Dignity” OR AB “Patient Dignity”

S3 TI "dignity with car*" OR AB "dignity with car*"

S2 TI “car* with dignity” OR AB “car* with dignity”

S1 TI “dignified car*” OR AB “dignified car*”

**Embase**

#33 #29 AND #30 AND #31 AND ([adult]/lim OR [young adult]/lim OR [middle aged]/lim OR [aged]/lim OR [very elderly]/lim) AND [embase]/lim AND [english]/lim

#32 #29 AND #30 AND #31

#31 #18 OR #19 OR #20 OR #21 OR #22 OR #23 OR #24 OR #25 OR #26 OR #27 OR #28

#30 #7 OR #8 OR #9 OR #10 OR #11 OR #12 OR #13 OR #14 OR #15 OR #16 OR #17

#29 #1 OR #2 OR #3 OR #4 OR #5 OR #6

#28 'patient reported outcome measure'/exp

#27 'cross sectional*': ab, ti

#26 survey*: ab, ti

#25 quantitative*: ab, ti

#24 measure*: ab, ti

#23 questionnaire*: ab, ti

#22 instrument*: ab, ti

#21 scale*: ab, ti

#20 tool*: ab, ti

#19 'patient reported experience measure*': ab, ti

#18 'patient reported outcome measure*': ab, ti

#17 'hospital care'/exp

#16 palliativ*: ab, ti

#15 postoperative: ab, ti

#14 preoperative: ab, ti

#13 oncolog*: ab, ti

#12 'clinical setting*': ab, ti

#11 ward*: ab, ti

#10 inpatient*: ab, ti

#9 'acute admission*': ab, ti

#8 hospital*: ab, ti

#7 'acute car*': ab, ti

#6 'human dignity'/exp

#5 'car* in dignity': ab, ti

#4 'patient dignity': ab, ti

#3 'dignity with car*': ab, ti

#2 'car* with dignity': ab, ti

#1 'dignified car*': ab, ti

**PsychInfo (via EBSCOhost platform)**

S33 S30 AND S31 AND S32

S32 S19 OR S20 OR S21 OR S22 OR S23 OR S24 OR S25 OR S26 OR S27 OR S28 OR S29

S31 S8 OR S9 OR S10 OR S11 OR S12 OR S13 OR S14 OR S15 OR S16 OR S17 OR S18

S30 S1 OR S2 OR S3 OR S4 OR S5 OR S6 OR S7

S29 DE "Patient Reported Outcome Measures"

S28 TI “Cross Sectional*” ORAB “Cross Sectional*”

S27 TI Survey* OR AB Survey*

S26 TI Quantitative* OR AB Quantitative*

S25 TI Measure* OR AB Measure*

S24 TI Questionnaire OR AB Questionnaire

S23 TI Instrument* OR AB Instrument*

S22 TI Scale* OR AB Scale*

S21 TI Tool* OR AB Tool*

S20 TI "Patient reported experience measure*" ORAB "Patient reported experience measure*"

S19 TI "Patient reported outcome measure*" OR AB "Patient reported outcome measure*"

S18 DE "Hospitalization" OR DE "Hospital Admission "OR DE "Hospital Discharge" OR DE "Psychiatric Hospitalization" OR DE "Hospitalized Patients" OR DE "Hospitals" OR DE "Psychiatric Hospitals"

S17 TI Palliativ* OR AB Palliativ*

S16 TI Postoperative OR AB Postoperative

S15 TI Preoperative OR AB Preoperative

S14 TI oncolog* OR AB oncolog*

S13 TI “clinical setting*” OR AB “clinical setting*”

S12 TI Ward* OR AB Ward*

S11 TI Inpatient* OR AB Inpatient*

S10 TI “Acute Admission*” OR AB “Acute Admission*”

S9 TI Hospital* OR AB Hospital*

S8 TI “Acute Car*” OR AB “Acute Car*”

S7 DE "Dignity"

S6 TI "car* in dignity" OR AB "car* in dignity"

S5 TI “Patient Reported Dignity” OR AB “Patient Reported Dignity”

S4 TI “Patient Dignity” OR AB “Patient Dignity”

S3 TI "dignity with car*" OR AB "dignity with car*"

S2 TI “car* with dignity” OR AB “car* with dignity”

S1 TI “dignified car*” OR AB “dignified car*”

Limiters: English Language; Date of Publication: 2000-2021; All Adult: 18+ years; Scholarly (Peer Reviewed) Journals; Search modes - Boolean/Phrase
